# Supplementary material for: Telomere Shortening and Psychiatric Disorders: A Systematic Review
Source: Cells. 2021 Jun 7;10(6):1423. doi: 10.3390/cells10061423 (PMC8227190; doi:10.3390/cells10061423)
Supplement: Supplementary file 1 [file cells-10-01423-s001.zip › Table S2.pdf]

Table S2

Quality assessment of cohort studies by the Newcastle-Ottawa Scale (NOS)

**Author**	**Selection**	**Comparability**	**Outcome**
Hoen et al.[21]	☆☆☆	☆☆	☆☆
Hoen et al.[23]	☆☆☆	☆☆	☆☆
Verhoeven et al.[25]	☆☆☆☆	☆☆	☆☆
Verhoeven et al.[26]	☆☆☆☆	☆☆	☆☆
Vance et al.[41]	☆☆☆☆	☆☆	☆☆☆
Starnino et al.[42]	☆☆☆	☆☆	☆
Solomon et al.[54]	☆☆	☆	☆☆☆
Verhoeven et al.[55]	☆☆☆	☆	☆☆☆
Malan et al.[60]	☆☆☆	☆	☆☆☆
