# Supplementary material for: Telomere Shortening and Psychiatric Disorders: A Systematic Review
Source: Cells. 2021 Jun 7;10(6):1423. doi: 10.3390/cells10061423 (PMC8227190; doi:10.3390/cells10061423)
Supplement: Supplementary file 1 [file cells-10-01423-s001.zip › Table S3.pdf]

Table S3

Quality assessment of cross-sectional studies by the adapted version of the Newcastle-Ottawa Scale (NOS)—see Appendixes

Author	Selection	Comparability	Exposure
Shaffer et al.[16]	☆☆☆☆	☆☆	☆☆☆
Zhang et al.[57]	☆☆	☆☆	☆☆
AlAhwal et al.[24]	☆	☆☆	☆☆
Needham et al.[11]	☆☆☆	☆☆	☆☆
Whisman et al.[27]	☆☆☆☆	☆☆	☆☆
Lin et al.[28]	☆☆	☆☆	☆☆☆
Zhao et al.[29]	☆☆☆	☆☆	☆☆☆
Chae at al.[31]	☆☆☆	☆☆	☆☆
Phillips et al.[32]	☆☆☆	☆	☆☆
Schaakxs et al.[35]	☆☆☆☆	☆☆	☆☆
Georgin-Lavialle et al.[37]	☆	☆	☆☆
Putermanet et al.[39]	☆☆	☆	☆☆☆
Wang et al.[43]	☆☆☆☆	☆☆	☆☆
Liu et al.[46]	☆☆☆☆	☆☆	☆☆
Wium-Andersen et al.[49]	☆☆☆☆	☆☆	☆☆☆
Huzen et al.[50]	☆☆☆	☆	☆☆☆
Zhang et al.[67]	☆☆☆	☆	☆☆
Bersani et al.[63]	☆	☆☆	☆☆
O'Donovan et al.[58]	☆	☆☆	☆☆
Kang et al.[59]	☆☆	☆☆	☆☆
Boks et al.[61]	☆☆	☆☆	☆☆
Roberts et al.[62]	☆☆	☆☆	☆☆☆
Ladwig et al.[63]	☆☆☆☆	☆☆	☆☆☆
Kim et al.[66]	☆☆☆	☆☆	☆☆
Groer et al.[69]	☆☆	☆☆	☆☆
