# Supplementary material for: Telomere Shortening and Psychiatric Disorders: A Systematic Review
Source: Cells. 2021 Jun 7;10(6):1423. doi: 10.3390/cells10061423 (PMC8227190; doi:10.3390/cells10061423)
Supplement: Supplementary file 1 [file cells-10-01423-s001.zip › Table S1.pdf]

Table S1

Quality assessment of case-control studies by the Newcastle-Ottawa Scale (NOS)

Authors	Selection	Comparability	Exposure
Tyrka et al.[18]	☆☆	☆☆	☆☆☆
Vincent et al.[25]	☆☆☆☆	☆	☆
Liu et al.[33]	☆	☆	☆☆
Wang X et al.[34]	☆☆	☆☆	☆☆
Wolkowitz et al.[36]	☆☆☆	☆☆	☆☆☆
Verhoeven et al.[38]	☆☆☆	☆☆	☆☆☆
Blom et al.[40]	☆☆	☆☆	☆☆
Wikgren et al.[44]	☆☆☆	☆	☆☆
Szebeni et al.[45]	☆	☆☆	☆
Boeck et al.[47]	☆☆☆	☆☆	☆☆
Jiménez et al.[48]	☆☆	☆☆	☆☆
Simon et al.[51]	☆☆☆	☆☆	☆☆
Harttman et al.[52]	☆☆	☆☆	☆☆
Karabatsiakis et al.[53]	☆☆☆	☆☆	☆☆
Mamdani et al.[56]	☆	☆	☆☆
Jergović et al.[64]	☆☆☆	☆☆	☆☆
Avetyan et al.[65]	☆☆☆	☆	☆☆☆
Verhoeven et al [68]	☆☆☆☆	☆☆	☆☆☆
Prelog et al.[70]	☆☆	☆☆	☆☆☆
